# Location of lateral tibial plateau fractures relative to the posterolateral complex predicts the need for extension of lateral approaches: A retrospective observational study

**DOI:** 10.1007/s00068-025-02933-4

**Published:** 2025-07-21

**Authors:** Michael J. Raschke, Elmar Herbst, Oliver Riesenbeck, Christoph Kittl, Christian Peez, J. Christoph Katthagen

**Affiliations:** Department of Trauma, Hand and Reconstructive Surgery, University Hospital Münster, Waldeyerstraße 1, 48149 Münster, Germany

**Keywords:** Tibial plateau fracture, Fracture type-specific approach selection, Extended lateral approaches, Lateral epicondyle osteotomy

## Abstract

**Purpose:**

Extended lateral approaches have been proposed to improve visualization and therefore reduction quality of the articular surface in lateral tibial plateau fractures. However, recommendations regarding the exact morphology of lateral tibial fractures requiring extended approaches are lacking.

**Methods:**

A retrospective observational cohort study was conducted using data from patients who underwent surgical treatment of a tibial plateau fracture involving the lateral tibial plateau (AO/OTA 41-B3 and 41-C3) at a level 1 trauma center between January 2020 and May 2024. Demographics, patient positioning, and surgical approaches were recorded. Comprehensive examinations on preoperative computed tomography (CT) scan were performed evaluating the morphology of lateral tibial plateau fractures relative to the posterolateral ligamentous structures.

**Results:**

143 patients (53.8% female, 46.2% male) with a mean age of 51.3 ± 14.3 years were included. Three distinct types of lateral tibial plateau fractures were identified. The most frequent fracture types observed were anterior to the posterolateral ligamentous structures (38.5%) and at level of the posterolateral complex (36.4%), followed by a fracture location posterior to the posterolateral ligamentous structures (25.1%). Extended lateral approaches using lateral femoral epicondyle osteotomy were performed in 17.5% of cases, with fractures posterior to the posterolateral ligamentous structures more likely to have an extended approach (80.0%, *p* < 0.001).

**Conclusions:**

Lateral tibial plateau fractures show three distinct fracture types, with the fracture location relative to the posterolateral ligamentous structures predicting extension of lateral approaches. For fractures extending posterior to the posterolateral complex, preoperative planning should include prone or lateral patient positioning and selection of an extended lateral approach.

**Level of evidence:**

III.

## Introduction

Tibial plateau fractures represent a severe injury to the knee joint accounting for 23 per 100.000 fractures annually, with the incidence steadily increasing [1–3]. These fractures commonly involve the lateral tibial plateau (isolated or combined), often with multifragmentary destruction of the articular surface [4–6]. Despite modern surgical developments, the treatment of these intra-articular fracture patterns remains challenging, as evidenced by clinical studies reporting unsatisfactory functional outcomes and high rates of conversion to total knee arthroplasty at short and mid-term follow-up [7–14]. Key factors to improve patient-reported outcomes and to reduce the risk of post-traumatic osteoarthritis are anatomic reconstruction of the articular surface and preservation of limb alignment, for which a thorough preoperative planning and proper selection of the surgical approach are essential [10, 11, 15, 16].

With an increased understanding of mechanism-dependent fracture patterns and the approach-related visualization of the tibial plateau [4, 5, 12, 16], individualized treatment strategies have been developed to further improve quality of fracture reduction [17]. However, articular malreduction following fracture fixation may still occur in up to 80% of cases [7, 8], primarily localized in the posterolateral quadrant of the tibial plateau [6, 8, 10]. Especially fractures of the postero-latero-lateral (PLL) and postero-latero-central (PLC) segments cannot be sufficiently exposed by using intraoperative fluoroscopy [18–20] or standard anterolateral and posterolateral approaches [16, 18, 21]. As the posterolateral ligamentous structures cover this part of the articular surface, extended lateral approaches by osteotomizing the lateral femoral epicondyle or fibular head have been established with improved short-term results [22–26]. However, lateral tibial plateau fractures are characterized by heterogenous fracture lines [4, 5], so that recommendations which fracture type requires an extended approach to achieve adequate fracture exposure are lacking.

The aim of this study was to investigate the location of lateral tibial plateau fractures in relation to the posterolateral ligamentous structures. It was hypothesized that the use of extended lateral approaches would increase with more posterior fracture location.

## Materials and methods

**Fig. 1:**
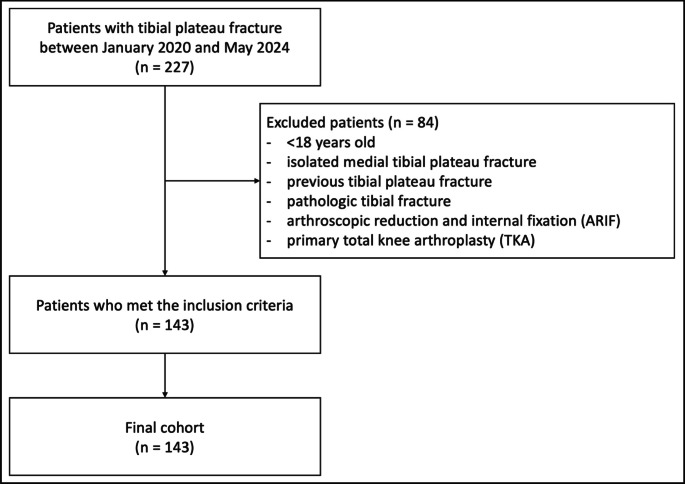
Flowchart of retrospective selection of the study population, consisting of patients with tibial plateau fracture who underwent open reduction and internal fixation at our department

A retrospective observational cohort study was conducted of all patient data from a Level 1 Trauma Center (Department for Trauma, Hand and Reconstructive Surgery, University Hospital Münster) who underwent open reduction and internal fixation (ORIF) of a tibial plateau fracture between January 2020 and May 2024. All patients with an uni-/bicondylar tibial plateau fracture, affecting the lateral tibial plateau (AO/OTA 41-B3 and 41-C3) were identified from the digital hospital documentation system and screened for eligibility. The following inclusion criteria were required: (1) age > 18 years, with closed physes; (2) complete medical records; (3) preoperative computed tomography (CT) scan confirming lateral tibial plateau involvement, with or without medial tibial plateau fracture; and (4) surgical treatment with ORIF by a leading senior orthopedic trauma knee surgeon (MJR, EH, CK and/or JCK). Patients with previous and isolated medial tibial plateau fractures, open fractures, arthroscopic-assisted reduction and internal fixation (ARIF), primary total knee arthroplasty (TKA), or pathologic fracture were excluded (Fig. 1).

The present study was reviewed and approved by the Institutional Ethics Committee of the University of Muenster (File number 2024-421-f-S).

### Patient-specific data

Age at primary surgery, sex, and mechanism of the accident were recorded. The fracture type was determined based on preoperative CT scans according to the AO/OTA fracture classification. The operative report was reviewed for patient positioning and surgical approaches used, with special emphasis on extension of the lateral approaches.

### Analysis of fracture morphology

**Fig. 2:**
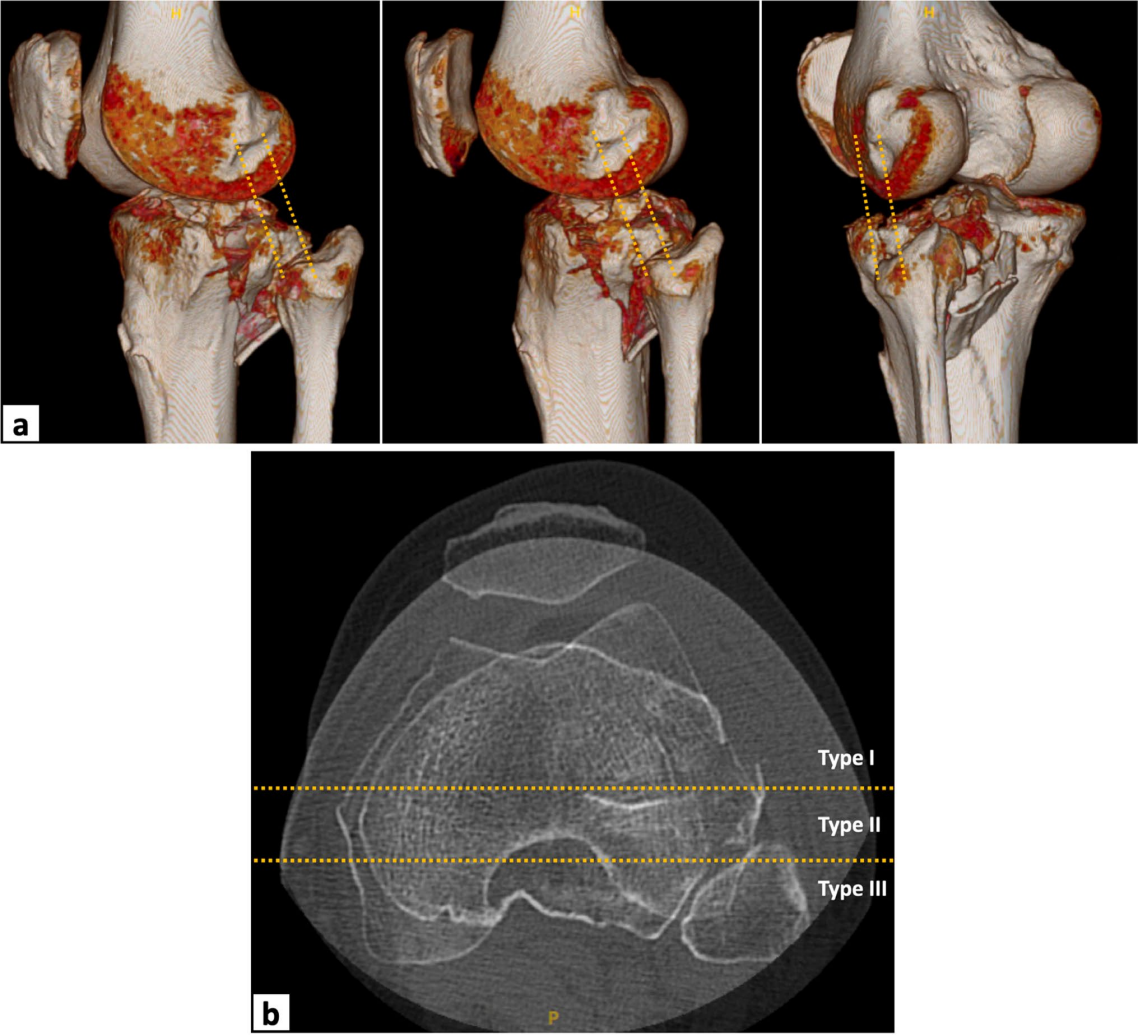
Schematic illustration of the fracture classification process. (**a**) Using the 3D reconstructions of the preoperative computed tomography (CT) scans, an imaginary line was drawn between the anterior and posterior aspect of the lateral epicondyle and the fibular tip, respectively, to assess the expansion of the depression zone of the lateral tibial plateau fracture relative to the anatomic course of the lateral collateral ligament (LCL). (**b**) Depending on the location of the depression zone, lateral tibial plateau fractures were classified into three different fractur types: Type I represent a lateral tibial plateau fracture with the main depression zone completely anterior to the LCL, whereas Type III fractures are characterized by a fracture zone posterior to the LCL. Type II fractures are borderline fractures characterized by location of the main depression zone at the level of the LCL

The 3D reconstructions and axial/sagittal planes of the preoperative CT scans were used to analyze the expansion of the depression zone of the lateral tibial plateau fracture relative to the PLC. An imaginary line was drawn between the anterior and posterior aspect of the lateral epicondyle and the fibular tip, representing the anatomical course of the LCL (Fig. 2a) [27, 28]. Based on this, lateral tibial plateau fractures were classified into three different fracture types. Type I fractures represent a lateral tibial plateau fracture with the fracture zone completely anterior to the LCL and without involvement of the posterior column, whereas Type III fractures are characterized by a fracture zone extending posterior to the LCL and involving the posterolateral segments (PLL and PLC). Subtype IIIa represents an extensive posterolateral impaction, whereas subtype IIIb represents an isolated fractures of the posterolateral rim or wall without involvement of the anterolateral segments (ALL and ALC). Type II fractures are borderline fractures characterized by the location of the fracture zone at the level of the LCL (Fig. 2b). The classification of the lateral tibial plateau fractures was performed and documented by a by a leading senior orthopedic knee surgeon (JCK).

### Statistical analysis

Statistical analysis was performed using Prism (Version 9, GraphPad Software). Categorical variables were reported as count and percentage. Continuous variables were presented as mean ± standard deviation. Data normality of continuous variables were tested and proved by using the Shapiro-Wilk test. Group comparison of categorical variables was performed with the Chi-square test or the Fisher’s exact test, as appropriate. Group comparison of continuous variables was performed with the Mann–Whitney U test or an unpaired t-test, as appropriate. Overall statistical significance was set to *p* < 0.05.

## Results

143 patients were included in the present study. The mean age within the cohort was 51.3 ± 14.3 years, with 77 (54.6%) female and 66 (45.6%) male patients. Unicondylar lateral tibial plateau fractures were observed in 62 patients (43.3%), while 81 patients (56.7%) had a bicondylar tibial plateau fractures. 59 patients (50.8%) suffered the tibial plateau fracture from a high-energy trauma mechanism, while 57 patients (49.2%) had a low-energy trauma. A detailed summary of the demographics can be found in Table 1.

**Table 1:** Descriptive statistics of the demographical and surgical data of the entire study group

Variable	Total study group
Number of included patients, *n*	143
Age (years)	51.3 ± 14.3 (20–79)
Sex	
Male, *n (%)*	66 (45.6%)
Female, *n (%)*	77 (54.4%)
Accident mechanism	
Fall < 3 m	38 (26.6%)
Fall > 3 m	21 (14.7%)
Motorcycle accident	23 (16.1%)
Car accident	25 (17.4%)
Bicycle accident	27 (18.9%)
Other	9 (6.3%)
Laterality	
Right, *n (%)*	72 (50.3%)
Left, *n (%)*	71 (49.7%)
Fracture type	
AO/OTA 41-B3, *n (%)*	62 (43.3%)
AO/OTA 41-C3, *n (%)*	81 (56.7%)
Patient positioning	
Supine position, *n (%)*	70 (48.9%)
Prone position, *n (%)*	38 (26.7%)
Lateral position, *n (%)*	35 (24.4%)
Extended lateral approach	
Yes, *n (%)*	25 (17.5%)
No, *n (%)*	96 (82.5%)
Fracture morphology	
Type I (anterior to PLC), *n (%)*	55 (38.5%)
Type II (at PLC level), *n (%)*	52 (36.4%)
Type III (posterior to PLC), *n (%)*	36 (25.1%)
- Type IIIa (extensive posterolateral impaction), *n (%)*	26 (13.9%)
- Type IIIb (posterolateral wall/rim fracture), *n (%)*	10 (11.2%)

Categorical variables are presented as count and percentage; continuous variables are presented as mean ± standard deviation. *PLC* posterolateral complex, *AO/OTA* Arbeitsgemeinschaft für Osteosynthesefragen/Orthopaedic Trauma Association

### Incidence and fracture morphology

**Fig. 3:**
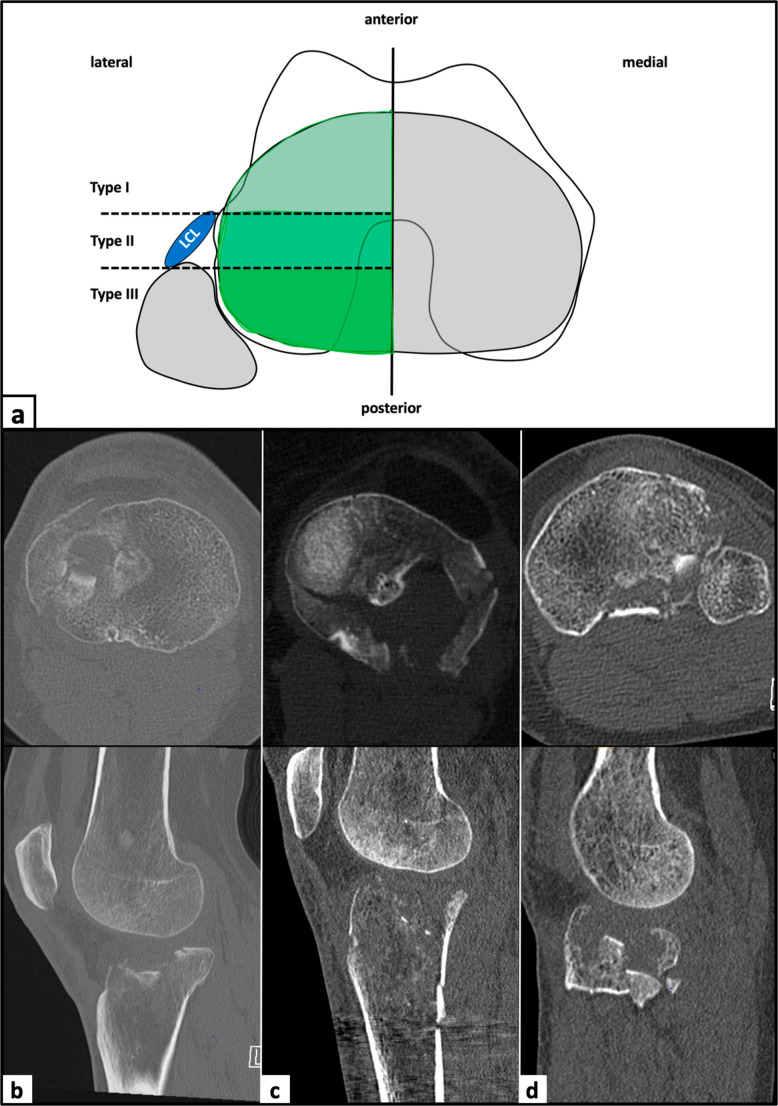
Classification of lateral tibial plateau fractures. (**a**) Schematic illustration of the three different fracture types. (**b**) Type I fracture, anterior to the ligamentous structures of the posterolateral corner. (**c**) Type II fracture, at the level of the posterolateral ligamentous structure. (**d**) Type III fracture, posterior to the ligamentous structures of the posterolateral corner

Three distinct types of lateral tibial plateau fractures were identified (Fig. 3). The most frequently observed fracture types were anterior to the posterolateral ligamentous structures (Type I; 38.5%) and at the level of the posterolateral complex (Type II; 36.4%), followed by a fracture location posterior to the posterolateral ligamentous structures (Type III; 25.1%).

### Extended lateral approach versus non-extended lateral approach

**Fig. 4:**
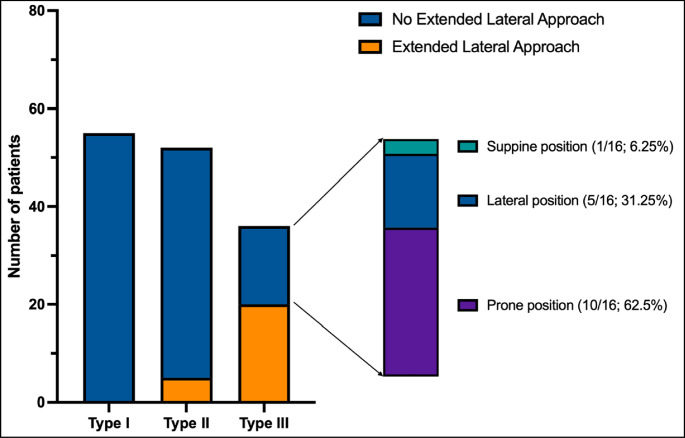
Group comparison (extended lateral approach versus non-extended lateral approach)

Extended lateral approaches by osteotomizing the lateral femoral epicondyle were used to reconstruct the lateral tibial plateau in 25 patients (17.5%), with 20 patients having a fracture posteriorly (Type III; 80.0%) and 5 patients at the level of the posterolateral ligamentous structures (Type II; 20.0%), respectively. Of the remaining 16 patients with Type III fractures who did not receive an extended lateral approach, 10 patients had pure posterior pathology of the posterior column involving the posterolateral wall or rim (Type IIIb) and were treated with posterior plate fixation in prone position only (62.5%). 87.3% of the type I fractures were treated with a standard anterolateral approach, and none of these fractures required an extension of the approach (Tables 2 and 3; Fig. 4).

**Table 2:** Descriptive statistics of fracture type-specific patient and plate positioning for the entire study group

Variable	Fracture type			
	Type I	Type II	Type IIIa	Type IIIb
Patient positioning				
Supine position, *n (%)*	46 (83.6%)	22 (41.5%)	2 (8.0%)	0 (0.0%)
Prone position, *n (%)*	4 (7.3%)	15 (28.3%)	9 (36.0%)	10 (100.0%)
Lateral position, *n (%)*	5 (9.1%)	16 (30.2%)	14 (56.0%)	0 (0.0%)
Plate positioning				
Anterolateral, *n (%)*	48 (87.3%)	21 (39.6%)	5 (20.0%)	0 (0.0%)
Posterolateral, *n (%)*	0 (0.0%)	3 (5.7%)	2 (8.0%)	10 (100.0%)
Anterolateral and posterolateral, *n (%)*	7 (12.7%)	29 (54.7%)	18 (72.0%)	0 (0.0%)

Categorical variables are presented as counts and percentages

**Table 3:** Group comparison (extended lateral approach versus non-extended lateral approach)

Variable	Extended lateral approach		*p* value
	Yes	No	
Number of patients, *n*	25 (17.5%)	118 (82.5%)	-
Age (years)	51.7 ± 13.1	51.1 ± 14.6	0.835
Sex			0.379
Male, *n (%)*	14 (56.0%)	53 (44.9%)	
Female, *n (%)*	11 (44.0%)	65 (55.1%)	
Fracture type			0.449
AO/OTA 41-B3, *n (%)*	10 (40.0%)	53 (44.9%)	
AO/OTA 41-C3, *n (%)*	15 (60.0%)	65 (55.1%)	
Patient positioning			**< 0.001**
Supine position, *n (%)*	5 (20.0%)	65 (55.1%)	
Prone position, *n (%)*	4 (16.0%)	34 (28.8%)	
Lateral position, *n (%)*	16 (64.0%)	19 (16.1%)	
Fracture morphology			**< 0.001**
Type I (anterior to PLC), *n (%)*	0 (0.0%)	55 (46.6%)	
Type II (at PLC level), *n (%)*	5 (20.0%)	47 (39.8%)	
Type III (posterior to PLC), *n (%)*	20 (80.0%)	16 (13.6%)	

Categorical variables are presented as count and percentage; continuous variables are presented as mean ± standard deviation

## Discussion

The most important finding of the present study was that lateral tibial plateau fractures have heterogenous fracture lines and variable extent of the depression zones. These fractures presented in three distinct types, with the fracture location relative to the posterolateral ligamentous structures predicting the need for extension of lateral approaches. Lateral femoral epicondyle osteotomies to extend lateral approaches were performed more frequently for fractures located posterior to the posterolateral complex, whereas fractures anterior to the posterolateral ligamentous structures did not require extended lateral approaches. Therefore, the type of fracture should be considered in preoperative planning and might help define the ideal patient positioning and surgical approach for anatomic fracture reduction.

Tibial plateau fractures are associated with a seven times higher risk of osteoarthritis compared to the uninjured population, resulting in unsatisfactory functional outcomes and high rates of conversion to total knee arthroplasty at 2- to 5-year follow-up [7–14]. In addition to the injury-related cartilage damage, the reasons for this increased risk may also be related to non-anatomic fracture reduction. It has been demonstrated that malreduction with residual intra-articular step-offs > 2.0 mm and coronal plane deformities > 5° were significantly associated with osteoarthritis at 4-year follow-up [9, 29], possibly caused by the elevated tibiofemoral contact forces and the accelerated articular cartilage degeneration in the affected compartment [10, 30, 31]. As intraoperative fluoroscopy does not provide adequate visualization of intra-articular steps less than 5 mm [19, 20], anatomic reduction by direct visualization should be the primary goals in the treatment of tibial plateau fractures.

However, articular malreduction after tibial plateau fracture fixation occurs primarily in the posterolateral quadrant of the tibial plateau in up to 80% of cases, with the PLL and PLC segments being the most commonly affected [6, 8, 10]. This has been attributed to the limited visualization of these segments by standard anterolateral and posterolateral approaches, as the posterolateral ligamentous structures cover this part of the articular surface [16]. In 2019, Krause et al. demonstrated in a quantitative anatomic study that the classic anterolateral approach only visualizes the anterior 36.6% of the articular surface, whereas a posterolateral approach provides 19.0% visualization of the posterior articular surface [16]. The exposure of the entire articular surface could be further increased to 65–80% when an osteotomy of the lateral epicondyle was performed [16, 32]. With this improved understanding of the proposed surgical approach-specific map of the lateral tibial plateau, the 3D morphology of the fracture pattern became the key determinant for preoperative planning and selection of an appropriate surgical approach to achieve anatomical fracture reduction [6, 17].

However, lateral tibial plateau fractures are characterized by heterogenous fracture lines and variable extent of the comminution zones [4, 33]. In the present study, these fractures presented in three distinct types, with the fracture location relative to the posterolateral ligamentous structures predicting the need for stepwise extension of the lateral approaches. In Type III fractures (posterior to the posterolateral ligamentous structures), 62% of patients were treated with a modified posterolateral approach as described by Frosch et al. combined with a lateral femoral epicondyle osteotomy to provide exposure of almost the entire lateral tibial plateau [16, 26, 32]. Due to the concept of a two-window dissection, this approach combines the visualization capabilities of the classic anterolateral and posterolateral approaches and provides direct access and manipulation of the posterolateral wall as well as the affected PLC and PLL segments. However, 10 of the remaining 16 patients with Type III fractures were treated with the aforementioned posterolateral approach in prone position without extension by osteotomizing the lateral femoral epicondyle, as in these cases only the posterolateral rim or wall was involved (Type IIIb), which can be adequately exposed through this approach [16]. Therefore, in cases of extensive depression fractures of the posterolateral segments (Type IIIa), extended posterolateral approaches in the lateral or prone position may be necessary for direct visualisation and intra-articular fracture reduction, whereas isolated fractures of the posterolateral column (Type IIIb) may not require a lateral femoral epicondyle osteotomy. Additionally, not only can improved visualization be achieved using this surgical approach, but so can reduction and the placement of implants. As fractures of the posterolateral segments are mainly caused by flexion-valgus trauma [4, 5, 12], these lateral-sided coronal plane fractures should be addressed with a proper posterolateral buttress plate, for which the aforementioned posterolateral approach again provides the necessary trajectory [16, 26, 32]. Otherwise, the osteosynthesis is likely to fail, as has been shown clinically [34, 35].

Conversely, all Type I fractures (anterior to the posterolateral ligamentous structures) did not require an extended lateral approach because the posterolateral ligamentous structures do not cover the fractured articular surface [16]. In these fractures, only the anterolateral quadrant and the anterior portions of the PLC and PLL segments need to be exposed, so we suggest performing the classic anterolateral approach, as the posterolateral approach is limited in its visualization of the antero-latero-central (ALC) segment and bears the risk of iatrogenic damage to the common peroneal nerve [16, 21, 26]. In addition, these fractures are primarily caused by extension-valgus trauma [4, 5, 12], so that a posterolateral buttress plate does not appear to be necessary to neutralize the resulting shear forces across the fracture site.

The Type II fractures (at the level of the posterolateral ligamentous structures) represent borderline entities, which are partially covered by the posterolateral ligamentous structures. With the aforementioned possibilities of a two-window dissection and the exposure of almost the entire lateral tibial plateau, a modified posterolateral approach as described by Frosch et al. is recommended in these cases, as this approach can be specifically adapted to the morphologic requirements of these fractures by stepwise extension with lateral epicondyle osteotomy and central meniscal subluxation [16, 22, 26, 32]. In addition, this approach offers the possibility to be place the plate osteosynthesis postero- and/or anterolaterally to adapt the proposed biomechanics of the osteosynthesis individually to the biomechanical requirements of the specific fracture type (flexion vs. valgus vs. valgus-flexion fracture) [4, 5, 12, 34, 35]. Therefore, we suggest performing the modified posterolateral approach in the lateral position for Type II fractures, as this approach allows for both anterolateral and/or posterolateral plate osteosynthesis as well as extension through a lateral femoral epicondyle osteotomy, if needed.

The present study has several limitations. First, comprehensive radiological and clinical outcomes as well as soft tissue management strategies are missing to validate the proposed concept and classification. Since this study was conducted to analyze the morphology of lateral tibial plateau fractures and the incidence of their subtypes to clarify the indications for a lateral epicondyle osteotomy, the collection and reporting of clinical data would have been beyond the scope of this study. Second, this study is subject to selection bias because only fractures treated by a group of surgeons from a single center were evaluated. Therefore, the results of this study represent an institutional philosophy rather than standardized recommendations. Nevertheless, the findings may have a positive impact on preoperative planning, as fracture location relative to the posterolateral ligamentous structures predicts selection of extended lateral approaches. Last, the present study focused only on the lateral tibial plateau and did not evaluate the treatment of concomitant medial plateau fractures. Nevertheless, complex lateral tibial plateau fractures often present with a coronal medial split fracture that can be treated with a separate direct posteromedial approach [16, 36, 37]. However, complex medial plateau fractures may complicate the preoperative planning and require individualized concepts, possibly with the need for intraoperative adjustment of patient positioning, e.g. change from prone to lateral position or the use of floppy position [38].

## Conclusion

Lateral tibial plateau fractures show three distinct fracture types, with the fracture location relative to the posterolateral ligamentous structures predicting extension of lateral approaches. For fractures located posterior to the posterolateral complex, preoperative planning should include proper prone or lateral positioning of the patient and selection of an extended lateral approach by osteomizing the lateral femoral epicondyle.

## Data Availability

No datasets were generated or analysed during the current study.

## Declarations

### Social Media Summary

Location of lateral tibial plateau fractures relative to the posterolateral complex was found to be an indicator for extension of lateral approaches @UK_Muenster

## References

[1] Bormann M, Neidlein C, Gassner C, Keppler AM, Bogner-Flatz V, Ehrnthaller C, Prall WC, Bocker W, Furmetz J. Changing patterns in the epidemiology of tibial plateau fractures: a 10-year review at a level-I trauma center. Eur J Trauma Emerg Surg. 2023;49(1):401–9. 10.1007/s00068-022-02076-w. 36057677 10.1007/s00068-022-02076-w

[2] Elsoe R, Larsen P, Nielsen NP, Swenne J, Rasmussen S, Ostgaard SE. Population-Based epidemiology of tibial plateau fractures. Orthopedics. 2015;38(9):e780–786. 10.3928/01477447-20150902-55. 26375535 10.3928/01477447-20150902-55

[3] Rupp M, Walter N, Pfeifer C, Lang S, Kerschbaum M, Krutsch W, Baumann F, Alt V. The incidence of fractures among the adult population of Germany–an analysis from 2009 through 2019. Dtsch Arztebl Int. 2021;118(40):665–9. 10.3238/arztebl.m2021.0238. 34140088 10.3238/arztebl.m2021.0238 PMC8727861

[4] Xie X, Zhan Y, Wang Y, Lucas JF, Zhang Y, Luo C. Comparative analysis of Mechanism-Associated 3-Dimensional tibial plateau fracture patterns. J Bone Joint Surg Am. 2020;102(5):410–8. 10.2106/jbjs.19.00485. 31855868 10.2106/JBJS.19.00485

[5] Yao X, Zhou K, Lv B, Wang L, Xie J, Fu X, Yuan J, Zhang Y. 3D mapping and classification of tibial plateau fractures. Bone Joint Res. 2020;9(6):258–67. 10.1302/2046-3758.96.Bjr-2019-0382.R2. 32728424 10.1302/2046-3758.96.BJR-2019-0382.R2 PMC7376308

[6] Krause M, Preiss A, Müller G, Madert J, Fehske K, Neumann MV, Domnick C, Raschke M, Südkamp N, Frosch KH. Intra-articular tibial plateau fracture characteristics according to the ten segment classification. Injury. 2016;47(11):2551–7. 10.1016/j.injury.2016.09.014. 27616003 10.1016/j.injury.2016.09.014

[7] Krause M, Preiss A, Meenen NM, Madert J, Frosch KH. Fracturoscopy is superior to fluoroscopy in the articular reconstruction of complex tibial plateau fractures-An arthroscopy assisted fracture reduction technique. J Orthop Trauma. 2016;30(8):437–44. 10.1097/bot.0000000000000569. 26978133 10.1097/BOT.0000000000000569

[8] Meulenkamp B, Martin R, Desy NM, Duffy P, Korley R, Puloski S, Buckley R. Incidence, risk factors, and location of articular malreductions of the tibial plateau. J Orthop Trauma. 2017;31(3):146–50. 10.1097/bot.0000000000000735. 27755337 10.1097/BOT.0000000000000735

[9] Parkkinen M, Madanat R, Mustonen A, Koskinen SK, Paavola M, Lindahl J. Factors predicting the development of early osteoarthritis following lateral tibial plateau fractures: mid-term clinical and radiographic outcomes of 73 operatively treated patients. Scand J Surg. 2014;103(4):256–62. 10.1177/1457496914520854. 24737855 10.1177/1457496914520854

[10] Rosteius T, Rausch V, Pätzholz S, Lotzien S, Königshausen M, Schildhauer TA, Geßmann J. Factors influencing the outcome after surgical reconstruction of OTA type B and C tibial plateau fractures: how crucial is the restoration of articular congruity? Arch Orthop Trauma Surg. 2023;143(4):1973–80. 10.1007/s00402-022-04405-5. 35303147 10.1007/s00402-022-04405-5 PMC10030527

[11] Singleton N, Sahakian V, Muir D. Outcome after tibial plateau fracture: how important is restoration of articular congruity?? J Orthop Trauma. 2017;31(3):158–63. 10.1097/bot.0000000000000762. 27984441 10.1097/BOT.0000000000000762

[12] Assink N, Vaartjes TP, Bosma E, van Helden SH, Ten Brinke JG, Hoekstra H, FFA IJ. Tibial plateau fracture morphology based on injury force mechanism is predictive for patient-reported outcome and conversion to total knee arthroplasty. Eur J Trauma Emerg Surg. 2024. 10.1007/s00068-024-02447-5. 38244051 10.1007/s00068-024-02447-5 PMC11249455

[13] Assink N, El Moumni M, Kraeima J, Bosma E, Nijveldt RJ, van Helden SH, Vaartjes TP, Ten Brinke JG, Witjes MJH, de Vries JPM, FFA IJ. Radiographic predictors of conversion to total knee arthroplasty after tibial plateau fracture surgery: results in a large multicenter cohort. J Bone Joint Surg Am. 2023;105(16):1237–45. 10.2106/jbjs.22.00500. 37196070 10.2106/JBJS.22.00500

[14] Olerud F, Garland A, Hailer NP, Wolf O. Risk of conversion to total knee arthroplasty after surgically treated tibial plateau fractures: an observational cohort study of 439 patients. Acta Orthop. 2024;95:206–11. 10.2340/17453674.2024.40605. 38712764 10.2340/17453674.2024.40605 PMC11075203

[15] Giannoudis PV, Tzioupis C, Papathanassopoulos A, Obakponovwe O, Roberts C. Articular step-off and risk of post-traumatic osteoarthritis. Evidence today. Injury. 2010;41(10):986–95. 10.1016/j.injury.2010.08.003. 20728882 10.1016/j.injury.2010.08.003

[16] Krause M, Krüger S, Müller G, Püschel K, Frosch KH. How can the articular surface of the tibial plateau be best exposed? A comparison of specific surgical approaches. Arch Orthop Trauma Surg. 2019;139(10):1369–77. 10.1007/s00402-019-03200-z. 31101980 10.1007/s00402-019-03200-z

[17] Frosch KH, Korthaus A, Thiesen D, Frings J, Krause M. The concept of direct approach to lateral tibial plateau fractures and Stepwise extension as needed. Eur J Trauma Emerg Surg. 2020;46(6):1211–9. 10.1007/s00068-020-01422-0. 32607776 10.1007/s00068-020-01422-0 PMC7691307

[18] Behrendt P, Berninger MT, Thürig G, Dehoust J, Christensen J, Frosch KH, Krause M, Hartel MJ. Nanoscopy and an extended lateral approach can improve the management of latero-central segments in tibial plateau fractures: a cadaveric study. Eur J Trauma Emerg Surg. 2023;49(3):1433–9. 10.1007/s00068-022-02188-3. 36484798 10.1007/s00068-022-02188-3 PMC10229462

[19] Haller JM, O’Toole R, Graves M, Barei D, Gardner M, Kubiak E, Nascone J, Nork S, Presson AP, Higgins TF. How much articular displacement can be detected using fluoroscopy for tibial plateau fractures? Injury. 2015;46(11):2243–7. 10.1016/j.injury.2015.06.043. 26199030 10.1016/j.injury.2015.06.043

[20] Mellema JJ, Doornberg JN, Molenaars RJ, Ring D, Kloen P. Tibial plateau fracture characteristics: reliability and diagnostic accuracy. J Orthop Trauma. 2016;30(5):e144–151. 10.1097/bot.0000000000000511. 27101164 10.1097/BOT.0000000000000511

[21] Behrendt P, Berninger MT, Thürig G, Dehoust J, Christensen JH, Frosch KH, Krause M, Hartel MJ. Anterolateral versus modified posterolateral approach for tibial plateau fractures with involvement of the posterior column: a cadaveric study. Eur J Trauma Emerg Surg. 2023;49(1):201–7. 10.1007/s00068-022-02113-8. 36171336 10.1007/s00068-022-02113-8 PMC9925589

[22] Korthaus A, Ballhause TM, Kolb JP, Krause M, Frosch KH, Hartel MJ. Extended approach to the lateral tibial plateau with central meniscal subluxation in fracture repair: feasibility and first clinical and radiographic results. Eur J Trauma Emerg Surg. 2020;46(6):1221–6. 10.1007/s00068-020-01467-1. 32865596 10.1007/s00068-020-01467-1 PMC7691302

[23] Krause M, Müller G, Frosch KH. [Extended medial and extended lateral approach for tibial plateau fractures]. Oper Orthop Traumatol. 2019;31(2):127–42. 10.1007/s00064-019-0593-9. 30887093 10.1007/s00064-019-0593-9

[24] Lobenhoffer P, Gerich T, Bertram T, Lattermann C, Pohlemann T, Tscheme H. [Particular posteromedial and posterolateral approaches for the treatment of tibial head fractures]. Unfallchirurg. 1997;100(12):957–67. 10.1007/s001130050218. 9492642 10.1007/s001130050218

[25] Bowers AL, Huffman GR. Lateral femoral epicondylar osteotomy: an extensile posterolateral knee approach. Clin Orthop Relat Res. 2008;466(7):1671–7. 10.1007/s11999-008-0232-5. 18373126 10.1007/s11999-008-0232-5 PMC2505238

[26] Frosch KH, Balcarek P, Walde T, Stürmer KM. A new posterolateral approach without fibula osteotomy for the treatment of tibial plateau fractures. J Orthop Trauma. 2010;24(8):515–20. 10.1097/BOT.0b013e3181e5e17d. 20657262 10.1097/BOT.0b013e3181e5e17d

[27] Crespo B, James EW, Metsavaht L, LaPrade RF. Injuries to posterolateral corner of the knee: a comprehensive review from anatomy to surgical treatment. Rev Bras Ortop. 2015;50(4):363–70. 10.1016/j.rboe.2014.12.008. 26401495 10.1016/j.rboe.2014.12.008 PMC4563052

[28] Moorman CT III, LaPrade RF. Anatomy and biomechanics of the posterolateral corner of the knee. J Knee Surg. 2005;18(2):137–45. 10.1055/s-0030-1248172. 15915835 10.1055/s-0030-1248172

[29] Rademakers MV, Kerkhoffs GM, Sierevelt IN, Raaymakers EL, Marti RK. Operative treatment of 109 tibial plateau fractures: five- to 27-year follow-up results. J Orthop Trauma. 2007;21(1):5–10. 10.1097/BOT.0b013e31802c5b51. 17211262 10.1097/BOT.0b013e31802c5b51

[30] Oeckenpöhler S, Domnick C, Raschke MJ, Müller M, Wähnert D, Kösters C. A lateral fracture step-off of 2 mm increases intra-articular pressure following tibial plateau fracture. Injury. 2022;53(3):1254–9. 10.1016/j.injury.2021.12.053. 35016775 10.1016/j.injury.2021.12.053

[31] Rosteius T, Rausch V, Jettkant B, Lotzien S, Schildhauer TA, Königshausen M, Geßmann J. Influence of articular step-off on contact mechanics in fractures of the posterolateral-central tibial plateau - a Biomechanical study. Knee. 2023;41:283–91. 10.1016/j.knee.2023.01.016. 36780843 10.1016/j.knee.2023.01.016

[32] Krause M, Frings J, Isik H, Frosch KH. Comparison of extended lateral approaches to the tibial plateau: the articular exposure of lateral epicondyle osteotomy with and without popliteus tendon vs. fibula osteotomy. Injury. 2020;51(8):1874–8. 10.1016/j.injury.2020.05.038. 32482428 10.1016/j.injury.2020.05.038

[33] Zhan Y, Zhang Y, Xie X, Luo C. Three-dimensional fracture mapping of multi-fragmentary patella fractures (AO/OTA 34C3). Ann Transl Med. 2021;9(17):1364. 10.21037/atm-21-1814. 34733916 10.21037/atm-21-1814 PMC8506535

[34] Kim CW, Lee CR, An KC, Gwak HC, Kim JH, Wang L, Yoon DG. Predictors of reduction loss in tibial plateau fracture surgery: focusing on posterior coronal fractures. Injury. 2016;47(7):1483–7. 10.1016/j.injury.2016.04.029. 27178768 10.1016/j.injury.2016.04.029

[35] Samsami S, Pätzold R, Winkler M, Herrmann S, Augat P. The effect of coronal splits on the structural stability of bi-condylar tibial plateau fractures: a Biomechanical investigation. Arch Orthop Trauma Surg. 2020;140(11):1719–30. 10.1007/s00402-020-03412-8. 32219572 10.1007/s00402-020-03412-8 PMC7557508

[36] Dehoust J, Münch M, Seide K, Barth T, Frosch KH. Biomechanical aspects of the posteromedial split in bicondylar tibial plateau fractures-a finite-element investigation. Eur J Trauma Emerg Surg. 2020;46(6):1257–66. 10.1007/s00068-020-01538-3. 33179130 10.1007/s00068-020-01538-3

[37] Eggli S, Hartel MJ, Kohl S, Haupt U, Exadaktylos AK, Röder C. Unstable bicondylar tibial plateau fractures: a clinical investigation. J Orthop Trauma. 2008;22(10):673–9. 10.1097/BOT.0b013e31818b1452. 18978541 10.1097/BOT.0b013e31818b1452

[38] Herbst E, Wessolowski MA, Raschke MJ. Extension of the medial approach to the tibial plateau via an osteotomy of the tibial insertion of the superficial medial collateral ligament. J Clin Med. 2023;12(16). 10.3390/jcm12165208. 10.3390/jcm12165208 PMC10455629 37629247

